# Bolingbroke House, Wandsworth Common

**Published:** 1888-06-23

**Authors:** 


### Bolingbroke House, Wandsworth Common.

A vast population, consisting largely of clerks and artisans, in the last ten years has settled around Clapham Junction. The nearest hospitals are four miles distant. Those hospitals, constantly appealing for funds, are intended for the *poor*, and not for middle-class folk, who do not wish to receive charity, and who are ready to pay according to their means for that skilled nursing and quietness which they cannot always obtain in their own homes. To meet their case, Bolingbroke House, Wandsworth Common, was opened seven years ago as a pay hospital. It has proved a great boon to nearly one hundred invalids in each year, about half of whom come from the adjacent parishes, and half from other parts of London and from the provinces. These patients have paid such sums as they could afford, averaging about a pound a-week. The cost of their treatment has been from thirty to fifty shillings per week, and the deficiency has been made up by the donations of sympathetic benevolence.

It was hoped at the outset that there would have been a sufficient number of wealthier patients, paying more than their actual cost, to balance the loss on those who paid less than their cost. This hope has not been fulfilled, owing mainly to the spread of the paying system in the more central parts of London. The result is that Bolingbroke House is doing its really beneficient work at an annual loss, which seriously threatens its continuance. It is felt that, with a middle-class population increasing, and apparently likely to increase in the districts around it with unexampled rapidity, it would be deplorable to surrender the hospital now that it is in perfect working order and has gained much publicity in the seven years of its existence.

The house is a thoroughly good hospital of twenty-five beds, with ground and capacity for ample extension. It is in a peculiarly healthful situation facing Wandsworth Common. In this year eighty-nine patients have paid no less than £926, but their treatment has involved a loss of about £250. The patients and their friends have testified their grateful appreciation of the boon which the house has been to them, in that it has spared them from resorting to the inevitable publicity and trying associations of a general hospital. Among the patients there were sixteen professional men, ten clerks, and nine governesses.

A comparatively small subscription list would enable the committee to maintain the hospital, which is most efficiently administered, and which they invite anyone to visit.

Further particulars will be given, and any donations gratefully acknowledged, by the resident medical officer, Dr. Cecil C. R. Lyster, Bolingbroke House, Wandsworth Common.

